# An evaluation of classification systems for stillbirth

**DOI:** 10.1186/1471-2393-9-24

**Published:** 2009-06-19

**Authors:** Vicki Flenady, J Frederik Frøen, Halit Pinar, Rozbeh Torabi, Eli Saastad, Grace Guyon, Laurie Russell, Adrian Charles, Catherine Harrison, Lawrence Chauke, Robert Pattinson, Rachel Koshy, Safiah Bahrin, Glenn Gardener, Katie Day, Karin Petersson, Adrienne Gordon, Kristen Gilshenan

**Affiliations:** 1 Mater Mothers' Research Centre, Mater Health Services, Brisbane, Australia; 2 Department of Obstetrics and Gynecology, University of Queensland, Brisbane, Australia; 3 Department of Genes and Environment, Division of Epidemiology, Norwegian Institute of Public Health, Oslo, Norway; 4 Department of Obstetrics, Gynecology and Reproductive Biology, Harvard Medical School, Boston, MA, USA; 5 Department of Pathology and Laboratory Medicine, Brown University Medical School, Providence, RI, USA; 6 Department of Midwifery, Faculty of Nursing Education, Arkeshus University College, Lillestrøm, Norway; 7 Alberta Perinatal Health Program, Edmonton, Canada; 8 Division of Anatomical Pathology, Department of Laboratory Medicine & Pathology, University of Alberta Hospitals, University of Alberta, Edmonton, Canada; 9 Department of Paediatric and Perinatal Pathology, King Edward Memorial Hospital, Perth, Australia; 10 Department of Obstetrics and Gynaecology, University of Pretoria School of Medecine, Pretoria, South Africa; 11 Division of Family Health Development, Ministry of Health Malaysia, Putrajaya, Malaysia; 12 Department of Obstetrics, Karolinska University Hospital, Stockholm, Sweden; 13 Department of Neonatal Medicine, Royal Prince Alfred Hospital, Sydney, Australia

## Abstract

**Background:**

Audit and classification of stillbirths is an essential part of clinical practice and a crucial step towards stillbirth prevention. Due to the limitations of the ICD system and lack of an international approach to an acceptable solution, numerous disparate classification systems have emerged. We assessed the performance of six contemporary systems to inform the development of an internationally accepted approach.

**Methods:**

We evaluated the following systems: Amended Aberdeen, Extended Wigglesworth; PSANZ-PDC, ReCoDe, Tulip and CODAC. Nine teams from 7 countries applied the classification systems to cohorts of stillbirths from their regions using 857 stillbirth cases. The main outcome measures were: the ability to retain the important information about the death using the *InfoKeep *rating; the ease of use according to the *Ease *rating (both measures used a five-point scale with a score <2 considered unsatisfactory); inter-observer agreement and the proportion of unexplained stillbirths. A randomly selected subset of 100 stillbirths was used to assess inter-observer agreement.

**Results:**

*InfoKeep *scores were significantly different across the classifications (*p *≤ 0.01) due to low scores for Wigglesworth and Aberdeen. CODAC received the highest mean (SD) score of 3.40 (0.73) followed by PSANZ-PDC, ReCoDe and Tulip [2.77 (1.00), 2.36 (1.21), 1.92 (1.24) respectively]. Wigglesworth and Aberdeen resulted in a high proportion of unexplained stillbirths and CODAC and Tulip the lowest. While *Ease *scores were different (*p *≤ 0.01), all systems received satisfactory scores; CODAC received the highest score. Aberdeen and Wigglesworth showed poor agreement with kappas of 0.35 and 0.25 respectively. Tulip performed best with a kappa of 0.74. The remainder had good to fair agreement.

**Conclusion:**

The Extended Wigglesworth and Amended Aberdeen systems cannot be recommended for classification of stillbirths. Overall, CODAC performed best with PSANZ-PDC and ReCoDe performing well. Tulip was shown to have the best agreement and a low proportion of unexplained stillbirths. The virtues of these systems need to be considered in the development of an international solution to classification of stillbirths. Further studies are required on the performance of classification systems in the context of developing countries. Suboptimal agreement highlights the importance of instituting measures to ensure consistency for any classification system.

## Background

Globally, over 3 million babies are stillborn every year with the vast majority occurring in developing countries [1]. While less frequent in developed countries (<1% of births), the large contribution of stillbirth to overall perinatal deaths combined with static or increasing rates over the past decade [2] clearly demonstrates that stillbirth is a major public health problem in these settings.

Classification of stillbirths, predicated on systematic assembly, storage and retrieval of the underlying cause of death and/or other relevant important information, is accepted as a crucial step towards the goal of reducing the numbers of stillborn infants [2,3]. However, the use of suboptimal classification systems may lead to a loss of important information and contributes to a high proportion of unexplained deaths. These deaths may be interpreted as unavoidable thereby diminishing the potential of immediate and longer term prevention strategies including research to address knowledge gaps. The wide variation in the reported contribution of unexplained stillbirth from 15% [4] to 71% [5] has been attributed to the classification system [5,6], thoroughness of investigation and the definition used [7,8]. The value of any death classification system is closely aligned with its ability to identify the underlying causes of death and the key factor which started the chain of events leading to the death. However, assigning a single cause is often challenging (and often inappropriate) due to the complexity of the clinical situation within which the fetus dies [9]. Therefore, classification systems for stillbirths must capture both the underlying cause and also the often multiple important factors and combinations of factors associated with these deaths. Due to inadequacies of the International Classification of Diseases (ICD) [10] coding system for this purpose, clinicians and researchers have been considering ways of classifying stillbirths to better understand the aetiology and patterns of causation of stillbirth for more than two decades [11].

Stillbirths first became notifiable in Scotland in 1940 [12] and in 1954 the classification developed by Sir Dugald Baird and his colleagues in Aberdeen for the purpose of audit and surveillance was published [13]. Subsequently, numerous systems have emerged. In a recent search we identified 33 new systems [4,5,8,9,11,13-40] and a further 12 modifications of these systems [5,41-51] for the classification of causes and associated conditions and/or suboptimal care among stillbirths. While the majority of these systems were designed for both stillbirths and neonatal deaths, three systems were designed specifically for stillbirths [4,28,33]. Two other systems, in addition to neonatal deaths, also included postneonatal deaths; one up to hospital discharge [19] and the other up to 12 months of age [26]. While it is important to analyse the causes of perinatal death according to its components of stillbirth and neonatal death [52], a system specifically designed to incorporate both groups enables interpretation of differences in the rates and causation across regions arising from variation in definition, reporting and registration practices for perinatal deaths [52].

According to Whitfield, the purpose of classification is 'to identify deficiencies in the provision of care, to focus attention where improvements are already possible and to indicate where new developments or knowledge may be expected to lead to further advance' [15]. The overarching goal, common to all classification systems, is the reduction of stillbirths and the primary purpose of classification, also common to all, is to conserve the useful information about the death. The secondary purpose relates to the intended use of the conserved information, which varies widely. There are four main categories: 1) to enable regional and international comparisons; 2) to undertake epidemiology and health surveillance; 3) for clinical practice i.e. quality improvement and parent counselling; and 4) for research use. These four categories represent very different requirements for a classification depending on the setting, e.g. a rural region of Africa compared to a tertiary teaching hospital in Europe. Despite the differences in use, the original case information is the data source for all classifications. For some this will be an extensive protocol of clinical history, examinations and tests. For others only sparse clinical information is available. Irrespective of the completeness of the original case information, the narrative of the case history is often a crucial part of the information that needs to be conserved. In addition to information capture, ease of use and inter-rater agreement are important requirements of any classification system.

A uniform global approach to classification of stillbirths is the ideal. The current use of disparate and possibly suboptimal classification systems for stillbirths limits the potential for advancements in the understanding of stillbirth and prohibits meaningful comparisons across regions and countries to assist in identifying priorities for prevention. There have been no studies evaluating the different contemporary classifications for stillbirths over a range of different users and settings focusing on the important virtues of information retention, ease of use and inter-rater agreement. We undertook this study to address this knowledge gap for the purpose of informing the development of an internationally accepted approach to the classification of stillbirths.

## Methods

### Identification of classification systems

We searched for published and unpublished reports of new classification systems or major revisions of existing systems which were developed for the classification of stillbirths. We restricted the search to the English language and searched electronic databases (Medline, Cochrane Library 1996–2006) and websites of relevant professional organisations. We also contacted expert informants and cross-referenced identified publications to identify relevant publications. As we were interested in classification systems that can be used widely, not only for detection of suboptimal care, but also to classify the main factors involved in a perinatal death as ascribed by an experienced clinical team, we excluded classification systems focusing on suboptimal care or avoidable factors and automated computer classification systems. In addition, we excluded reports of informal groupings of deaths, e.g. post hoc categorization based on the findings of hospital perinatal death committee meetings and duplicate publications of the same or very similar systems. For publications of the same classification system or those which were considered to be a minor modification, the most recent publication was chosen for inclusion.

The search identified 28 reports of potentially eligible systems. Following review of the full publications, 22 reports were excluded leaving six systems for evaluation: Amended Aberdeen [41]; Extended Wigglesworth [5], PSANZ-PDC (Perinatal Society of Australia and New Zealand – Perinatal Death Classification) [11,53], ReCoDe: Relevant conditions at death [4], Tulip [19], CODAC (Cause of Death and Associated Conditions) [18]. The exclusions were: systems focusing on suboptimal care [16,17,23,24,34]; automated computer systems [20,36]; duplicate publications (i.e. the same or very similar systems) [21,44,45,51,54-58]; studies reporting evaluations of systems [59-61]. Further, one system was excluded due to its major focus on postneonatal death conditions [26] and a further two were excluded as they were initial proposals of new systems [8,28], one of which has been subsequently published [62].

### Characteristics of included systems

Three systems are intended to be used in a strictly hierarchical manner (Aberdeen, Wigglesworth, ReCoDe), one system recommends a hierarchical approach to be used as a guide only (Tulip), another (PSANZ) also recommends a hierarchical approach as a guide apart from the initial category (congenital abnormalities) which takes priority. The remaining system (CODAC) uses a hierarchical approach for terminations of pregnancy only. Three classifications are intended to identify a single underlying cause of death (Tulip, Wigglesworth, Aberdeen), two aim to identify the cause of death (COD), if present, and associated conditions in secondary and tertiary levels of the system (CODAC and PSANZ-PDC). The remaining system, ReCoDe, aims to identify relevant conditions including either the cause of death and/or other relevant conditions, with the ability to assign two codes. Apart from the Tulip and CODAC classifications, the included systems use largely clinically based categories with very few categories for placental pathology. The Tulip classification, in addition to identifying a single demonstrable pathophysiological cause for the death, was also designed to identify the mechanism and the origin of the mechanism of the death, e.g. if the cause of an intrauterine death was attributed to infection, multiorgan failure would be considered the mechanism of the death and intrauterine infection the origin of the mechanism. All systems were designed in a developed country setting. Only one system was developed exclusively for stillbirths (ReCoDe). The number of categories and subcategories vary widely (Additional file 1).

### Main outcome measures

#### 1. Retaining relevant information: *InfoKeep Score*

To measure the extent to which the classification teams agreed that important information to aid in the understanding of the death was conserved and was retrievable after classification, we used a scoring system; *InfoKeep*. *InfoKeep *was designed specifically for this study and consisted of a five point rating scale from 0 (Disagree) to 4 (Agree). Prior to application of this scale, the classification teams responded to the question as to whether important information to assist in understanding the circumstances of the death was identified in each case by responding either as *No, Somewhat *or *Yes *across ten potential information sources. The information source categories provided were: maternal history or health; fetal history or health; intrapartum events or conditions; autopsy results; placental histopathology; examination of the cord and membranes; cultures or other tests for infection; genetic testing; other tests or examinations; and other sources.

#### 2. Ease of application: *Ease Score*

The *Ease *scoring system, also developed for the purpose of this study, was made up of a five point scale to measure, after the cause of death and associated conditions were determined for each case, the extent to which the classification team agreed that it was easy to identify the relevant category in the classification system. The scores ranged from zero to four with zero indicating that it was not possible to identify the relevant category in the classification and four indicating a relevant category was very easily identified.

#### 3. Inter-observer reliability

Inter-observer reliability for the major categories of the classification systems was assessed using a randomly selected subset of 100 stillbirths from five study teams who agreed to participate in this aspect of the study. Inter-observer agreement beyond chance for the main classification categories was assessed using the unweighted kappa statistic.

### Secondary outcome

The overall proportion of unexplained stillbirths resulting from the application of each classification.

### Classification teams and stillbirth cohorts

Study investigators made up the classification teams. As this study was designed to test how each system performed in a "real life" classification situation, membership of the classification teams was intended to reflect usual procedures at each of the participating sites. To reduce the potential for bias, developers of any of the included classification systems were excluded from participation in the classification teams.

Nine classification teams across five developed countries and two developing country settings were included: three in Australia (Brisbane, Sydney, and Perth) and one team each in Norway, Canada, US, South Africa, Malaysia and Sweden. Membership of the teams usually included two persons with varying backgrounds including: obstetrics, maternal fetal medicine specialists, midwifery, neonatology and paediatrics, perinatal pathology, and a public health specialist. All classification teams were experienced in classification of stillbirths through their usual practice either at a hospital or regional level. All stillbirth cases included in this study had been previously reviewed and classified by a multidisciplinary committee, in which the classification team members participated, using the classification system routinely used in their practice. These classifications were as follows: Australia – the PSANZ-PDC; Malaysia – modified Wigglesworth; South Africa – a modification of Aberdeen similar to PSANZ-PDC; US – an informal pathological grouping system; Sweden – The Stockholm Classification of Stillbirth; Norway – ICD 10 and an earlier version of CODAC; and Canada – modified Wigglesworth. For this study, six teams contributed population-based cases and three contributed cases from individual institutions. The primary purpose of classification for the population-based cohorts was for epidemiological analyses to identify areas for prevention through practice and policy improvements. The purpose for two hospital-based teams was primarily for clinical audit aimed at practice improvement as well as contributing to epidemiological population-based data. The remaining hospital-based team classified stillbirths for the purposes of research focusing on placental pathology.

### Testing procedure

Each classification team was asked to identify a consecutive series of 100 stillbirths according to local definition from the routinely collected data for the most recent time period and to assemble the information which is usually reviewed for each case. Eight teams used between 86 and 106 cases each and one team used 67 cases for testing, giving a total of 857 cases of stillbirths. Classification teams applied all six included classifications systems and assigned the two rating scores (*InfoKeep *and *Ease*) to each stillbirth from their own cohort.

Classification instructions, which were available in the public domain, were provided to the classification teams as well as paper-based classifications in a standardised format (Additional file 2). Teams were asked to become familiar with the instructions for each system prior to commencing the testing. As the instructions for the number of classification categories which could be assigned to each case varied across the classification, for consistency, the classification teams were asked to assign up to three categories for complicated cases. The written instructions provided for CODAC were not, at that time, available in the public domain. Five systems were used as paper-based systems and one, CODAC, in an electronic format. Four members of the research team independently applied the six classifications to a random sample (stratified by centre) of 100 stillbirths from five centres, to enable assessment of inter-rater agreement. For this analysis, deidentified case summaries from five centres were provided to the main coordinating centre for compilation and distribution to the testers.

### Data collection, management and analysis

Following review of each stillbirth case, the classification teams assigned the six classifications and the scoring systems using a purpose built database developed in Microsoft Excel. Following completion of the testing, the data file was sent electronically to the main coordinating centre for analysis using StataSE 9.2. The data analyst (KG) was not involved in either the development of any of the included classifications or any other aspect of the design or conduct of the study. Classification scores *InfoKeep *and *Ease *were analysed using ANOVA. Mean scores <2 were considered unsatisfactory. *InfoKeep *scores were analysed for the information categories in which teams responded as either *Yes *or *Somewhat *that significant information was identified. The level for rejection of a false positive finding was set at *p *= 0.05 or less for all outcome measures. Post hoc analysis was undertaken using the Bonferroni technique. To assess how each system performs for common stillbirth scenarios, subgroup analyses were performed for *InfoKeep *according to the presence of fetal growth restriction, placental pathology, congenital abnormality, intrapartum deaths and multiple pregnancies. These subgroups were assembled by combining all stillbirths classified into one or more of the relevant categories across the classifications systems. Data from developing country teams were compared with that from developed countries. In addition, the proportion of unexplained stillbirths was analysed across the classifications and classification teams. Inter-observer agreement beyond chance for the main classification categories was assessed using the unweighted kappa statistic with the following interpretation: poor <0.40; fair 0.40–<0.55; good 0.55–<0.70; very good 0.70–<0.85; and excellent ≥0.85 [63].

This study was approved by the Human Research Ethics Committee, Mater Health Services, Brisbane, Australia.

## Results

### Stillbirth characteristics

Of the 857 stillbirths included in the study, 256 (29.9%) were intrapartum deaths and 56 (7.6%) were from a multiple pregnancy. Placental pathology was classified by one or more of the classification systems in 506 (59.0%), fetal growth restriction in 168 (19.6%), and congenital abnormalities in 156 (18.2%).

### Information retention

**Figure 1:**
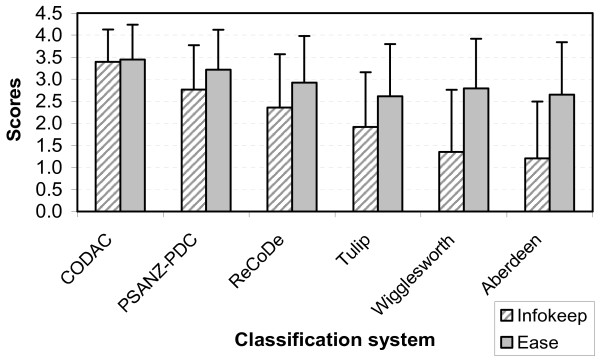
**Retention of important information (*InfoKeep*) and ease of use (*Ease*) scores by classification systems**. The extent to which the classification teams agreed that important information was conserved by the classification systems and was retrievable after classification was assessed using a scoring system (*InfoKeep*) consisting of a five point rating scale from 0 (Disagree) to 4 (Agree). Similarly, the extent to which the classification team agreed that it was easy to identify the relevant category in the classification system was assessed using the five point scoring system, *Ease*. Results represent the mean (± 1 SD) of the combined scores from each team for *InfoKeep *and *Ease *across the classifications systems.

Mean overall *InfoKeep *scores were significantly different across the classifications (p < 0.01). Post hoc analysis revealed that the difference was due to low scores for the Wigglesworth and Aberdeen classifications [mean (SD); 1.35 (1.41) and 1.21 (1.29) respectively]. CODAC received the highest scores [3.40 (0.73)] followed by PSANZ-PDC, ReCoDe and Tulip [2.77 (1.00), 2.36 (1.21) and 1.92 (1.24) respectively] (Figure 1).

Four classification systems received unsatisfactory mean *InfoKeep *scores (<2) for one or more of the 10 information categories. Aberdeen and Wigglesworth received unsatisfactory scores for all 10 information categories. Tulip received unsatisfactory scores for five categories [mean (SD)]: maternal history and health, 1.88 (1.64); autopsy 1.99 (1.41); cord and membranes, 1.81 (1.34); infections, 1.97 (1.47); and other tests, 1.70 (1.60). ReCoDe received a single unsatisfactory score for genetic testing [1.38 (1.50)].

### Ease of use

*Ease *scores were significantly different across the classifications (p < 0.01). However, all classifications received satisfactory scores. CODAC received the highest score [mean (SD)] 3.45 (0.79) followed by PSANZ-PDC 3.21 (0.91), ReCoDe with 2.92 (1.06), Wigglesworth 2.80 (1.19), Aberdeen 2.65 (1.18), and Tulip 2.61 (1.12) (Figure 1). Post hoc analyses revealed that ReCoDe and Wigglesworth were similar (*p *= 0.32), as were Aberdeen and Tulip (*p *= 1.0) and Aberdeen and Wigglesworth (*p *= 0.25).

### Inter-rater agreement

Inter-rater agreement for the major categories of the classifications across four classifiers using 100 stillbirth cases showed Aberdeen and Wigglesworth to have poor agreement with kappas of 0.35 and 0.25 respectively. Tulip performed best with a kappa of 0.74. The remainder had good to fair agreement as follows: CODAC, 0.65; PSANZ-PDC, 0.63; and ReCoDe, 0.51.

### Unexplained stillbirths

While comparison of the proportion of unexplained stillbirth yielded by each of the classification systems is extremely problematic due to the differences in available categories and approaches to classification, Wigglesworth and Aberdeen were shown to have the highest proportion of unexplained stillbirths (50.2% and 44.3% respectively) and CODAC the least (9.5%), while Tulip performed similarly to CODAC with 10.2% of cases unexplained. Including only the subcategory of unexplained stillbirth without evidence of utero-placental insufficiency (i.e. placental examination or antenatal Doppler evidence), PSANZ-PDC was shown to have a similar proportion of unexplained stillbirths as ReCoDe (15.4% and 13.8% respectively). Variation in the proportion unexplained was shown across the classification teams reflecting differences in interpretation of the classification systems. However, these differences were not significant (*p *= 0.38) (Additional file 3).

### Subgroup analyses

#### Information categories

**Figure 2:**
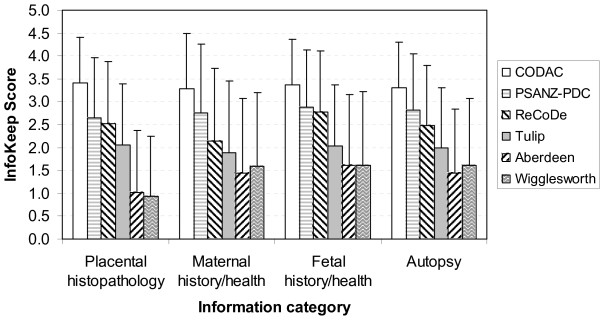
***InfoKeep *scores by the main sources of important information**. Prior to application of *InfoKeep*, the classification teams responded to the question as to whether important information to assist in understanding the circumstances of the death was identified in each case according to pre-defined information sources. Results represent the mean (± 1 SD) of the combined scores from each team for the most frequently reported information categories across the classifications system.

The most frequently reported categories where important information about the stillbirth was identified by the teams were: placental histopathology (62%); maternal history or health (59%); fetal history or health (39%); and autopsy results (34%). Significant information was identified to a lesser extent for the remaining categories as follows: cord and membranes (22%); intrapartum factors and events (21%); cultures or other tests for infections (11%); other tests or examinations (11%); genetic testing (6%); and other sources (4%). When examining the subgroups of the top four categories of important information sources, *InfoKeep *scores were similar to the overall analysis with Wigglesworth and Aberdeen receiving unsatisfactory scores and CODAC receiving the highest scores. The range of scores across the systems were as follows [mean (SD)]: CODAC, 3.28 (1.15) to 3.41 (1.01); PSANZ-PDC, 2.65 (1.32) to 2.89 (1.23); ReCoDe, 2.15 (1.59) to 2.78 (1.34); Tulip, 1.88 (1.64) to 2.06 (1.32); Aberdeen, 1.01 (1.37) to 1.62 (1.54); and Wigglesworth, 0.93 (1.32) to 1.62 (1.46) (Figure 2).

#### Stillbirth characteristics

**Figure 3:**
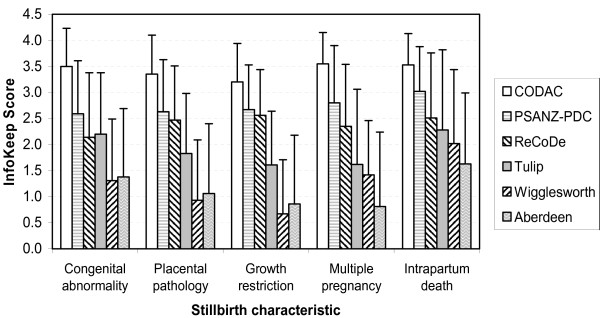
***InfoKeep *scores by stillbirth characteristics**. Analyses were undertaken to determine *InfoKeep *scores according to important subgroups according to stillbirth characteristics. Results represent the mean (± 1 SD) of the combined scores for these subgroups from each team *InfoKeep *scores across the classification systems.

Subgroup analyses were performed according to the presence of certain conditions as follows: fetal growth restriction (n = 168); placental pathology (n = 506); congenital abnormalities (n = 156); intrapartum death (n = 256); and multiple pregnancy (n = 56). The analyses revealed similar findings to the overall analysis. Aberdeen received unsatisfactory *InfoKeep *scores across all stillbirth subgroups. Wigglesworth received slightly higher scores for intrapartum deaths resulting in a satisfactory score [2.02 (1.54)]. CODAC consistently received the highest scores. Post hoc analysis revealed that PSANZ-PDC and ReCoDe performed similarly in three of the five stillbirth subgroups (fetal growth restriction, placental pathology and multiple pregnancy). Aberdeen and Wigglesworth performed similarly for the categories of fetal growth restriction, placental pathology, congenital abnormality and multiple pregnancy. The range of scores [mean (SD)] were as follows: CODAC, 3.55 (0.60) to 3.20 (0.74); PSANZ-PDC, 3.02 (0.86) to 2.59 (1.00); ReCoDe, 2.56 (0.88) to 1.92 (1.24); Tulip, 2.36 (1.64) to 1.61 (1.32); Aberdeen, 1.63 (1.42) to 0.81 (1.04); and Wigglesworth, 2.02 (1.54) to 0.67 (1.03) (Figure 3).

All systems received satisfactory *Ease *scores with the ranking by score unchanged. Tulip received the lowest *Ease *scores most frequently (three of the five subcategories) for [mean (SD)]: fetal growth restriction, 2.31 (1.14); placental pathology, 2.36 (1.08); and multiple pregnancy, 2.39 (1.37).

#### Developing country settings

Two developing country teams provided information on a total of 181 stillbirth cases. When compared to developed country cohorts, the frequency of information source categories from the developing country cohorts varied markedly and were statistically significantly different apart from one source; fetal history and health (35.2% developing versus 39.5% developed, *p *= 0.30). The frequency of the five main information categories for developing countries compared with that of developed settings were: maternal history and health 93.3% versus 50.3%; intrapartum events and conditions 41.9% versus 15.1%; placental histopathology 23.5% versus 71.8%; other tests 22.4% versus 8.1%. Minimal important information was identified from the remaining information categories (i.e. cultures, genetic testing). No autopsy examinations were performed in the cases from the developing countries (Table 1).

**Table 1:** Information sources, by country setting

	**Country setting**			
**Information source**	Developed n = 676	Developing n = 181	p-value	Overall

Maternal history/health	50.3%	93.3%	<0.01	59.3%
Fetal history/health	39.5%	35.2%	0.30	38.6%
Intrapartum events or conditions	15.1%	41.9%	<0.01	20.7%
Autopsy results	42.6%	0.00%	<0.01	33.7%
Placental histopathology	71.8%	23.5%	<0.01	61.6%
Cord and membranes	27.1%	2.2%	<0.01	21.9%
Cultures or other tests for infection	13.3%	3.9%	<0.01	11.4%
Genetic testing	7.3%	1.1%	<0.01	6.0%
Other tests	8.1%	22.4%	<0.01	11.1%
Other sources	3.6%	7.8%	0.02	4.4%

When compared to developed countries, *InfoKeep *scores were significantly higher for the developing country subgroup. The ranking of the classifications by *InfoKeep *score remained virtually unchanged from the overall analysis and was similar between the two subgroups. Wigglesworth performed better than Aberdeen in the developed country subgroup resulting in a satisfactory overall score (mean 2.47, SD 1.57). *Ease *scores were significantly higher for all classifications in the developing country subgroup with the exception of Aberdeen which was similar to that of the developed country subgroup (Table 2).

**Table 2:** *InfoKeep *and *Ease *scores, developed compared with developing country setting

	**Country setting**			
**Classification**	***InfoKeep*** **Mean (SD)**		***Ease*** **Mean (SD)**	

	Developed n = 676	Developing n = 181	Developed n = 676	Developing n = 181

CODAC	3.31 (0.77)	3.70 (0.43)	3.34 (0.84)	3.83 (0.40)
PSANZ-PDC	2.56 (1.00)	3.49 (0.59)	3.13 (0.95)	3.53 (0.62)
ReCoDe	2.31 (1.11)^±^	2.52 (1.49)^±^	2.86 (1.09)	3.20 (0.85)
Tulip	1.84 (1.07)	2.18 (1.69)	2.49 (1.12)	3.25 (0.93)
Wigglesworth	1.01 (1.16)	2.47 (1.57)	2.69 (1.24)	3.20 (1.07)
Aberdeen	1.12 (1.18)	1.48 (1.58)	2.62 (1.11)^¶^	2.85 (0.85)^¶^

^¶ ^*p *= 0.09; ^± ^*p *= 0.04, all other comparisons (i.e. developed vs. developing by classification) *p *= <0.01

#### Classification teams

Three classification teams worked in the same institution as developers of two of the included classifications: the Brisbane team with a developer of the PSANZ-PDC (VF) and the Norwegian and the US teams with CODAC (JFF, HP respectively). To explore the effect of potential bias on the results we examined *Ease *and *InfoKeep *scores assigned by these teams to those systems. Two of the three classification teams scored the classifications with which they were associated lower than did other classification teams. For PSANZ-PDC assigned by the Brisbane team, the overall *Ease *and *InfoKeep *scores [mean (SD)] were lower than the rest; *Ease *PSANZ-PDC 2.76 (0.99) versus the rest 3.27 (0.88), *InfoKeep *2.32 (1.07) versus 2.82 (0.98) (*p *< 0.01). This finding held when examining the three Australian teams combined versus the rest. The Norwegian and US scores for CODAC were not significantly different for *Ease *however (while the difference was small) *InfoKeep *was scored significantly higher than the rest; 3.55 (0.69) versus 3.35 (0.74) (*p *< 0.01).

## Discussion

The basic requirements for classification systems for stillbirth are to record the underlying cause of death and other relevant information to aid in understanding of the true contributors to stillbirth, to be easy to apply, and to perform robustly across different settings and classifiers. According to these criteria, this evaluation, by nine experienced international classification teams of six contemporary classifications across developed and developing country settings, did not identify any single system as clearly superior for stillbirths for the outcomes studied. However, CODAC performed consistently better than the rest in terms of information retention and ease of use and was also shown to have good inter-rater agreement. The Extended Wigglesworth and the Amended Aberdeen classifications were shown to be clearly inferior for all outcomes in this study. This result is consistent with the finding of others [5,6] which report that Wigglesworth and Aberdeen result in a large proportion of unexplained stillbirths. CODAC and PSANZ-PDC were the only classifications receiving satisfactory scores for information retention, across all sources of important information identified. ReCoDe received only one unsatisfactory score for information retention in the area of genetic testing which was shown to be one of the less frequent sources of important information. As would be expected, these systems also resulted in a low proportion of unexplained stillbirths. The numbers of available categories probably influenced the scoring for information retention as the two highest scoring systems (CODAC and PSANZ-PDC) also had the greater numbers of available categories. Other aspects of CODAC which may have resulted in better performance include its structured approach to identifying the underlying cause and its ability to capture narrative aspects of the death through the clustering of subcategories of the relevant associated conditions. The main sources of important information about the stillbirth came from the placenta, the maternal and fetal health and history and the autopsy. Three systems, CODAC, PSANZ-PDC and ReCoDe, performed satisfactorily in terms of retaining this information with CODAC again receiving the highest scores. Placental pathology was identified as an important source of information about the death in over 60% of stillbirths, consistent with other studies [54,61,64]. An evaluation of seven classification systems for stillbirths was recently reported by the developers of Tulip in the Netherlands [61]. This evaluation, which focussed on placental histopathology, found that Tulip performed best in retaining placental information and as a result reduced the proportion of unexplained stillbirths when compared with other stillbirth classifications. While our study did not confirm Tulip's superiority in this respect, comparison with this study is problematic due to differences in setting, investigation level, purpose and methodology. However, in our study Tulip resulted in a low proportion of unexplained stillbirths. This finding may result from the inclusion of an *unclassifiable *category for cases where it is deemed that important information was missing (e.g. adequate clinical history, autopsy or placental pathology). As placental pathology is an important finding in many stillbirths, we agree with Korteweg et al that further definition of the placental causes of stillbirth is needed and that further research to investigate the clinical manifestations of these placental causes of stillbirth is important in the prevention of these deaths.

The variation in the proportion of unexplained stillbirths across classifications is at least partly explained by the classification system. In the PSANZ system the unexplained group includes those with placental disease but who do not have growth restriction or other features. Other systems ignore growth restriction as an important factor and some ignore cases that are "unexplored" due to suboptimal investigations or where other important information is missing. Our study used materials from both developed and developing countries with varying investigation levels across the cohorts. In the participating developing countries, autopsy was not performed for any stillbirth whereas for developed countries the autopsy rate ranged from 40–80% and placental pathology rates ranged from 40–98%. Classification systems may perform quite differently in developing versus developed country settings due to dissimilar aetiologies of stillbirth [65] and the frequent paucity of information about the death in developing country settings. In this study, the information sources which were deemed important for stillbirths from developing country settings highlighted these differences. The placenta was an important source of information in just under one quarter of stillbirths in developing countries compared with 72% in the developed country cohorts as it was not often examined in developing countries. Maternal history was the source of important information in over 90% of cases from the developing country versus 50% in the developed countries. Important information relating to intrapartum events was identified in 42% of cases from the developing countries versus 15% in developed country settings. Despite these differences (which were likely to be largely as a result of differences in investigation level) the findings from developing country cohorts were very similar to those of the developed countries in terms of information retention and ease of use. Wigglesworth was shown to perform slightly better in terms of information retention in the developing countries than in developed countries. This may relate to a match between the lack of detail on stillbirths in these settings and the limited choice of categories in this system. The original [9] and modified Wigglesworth [5] systems have been the most frequently used classifications in developing country settings and have been found to be easy to apply and helpful in comparisons across countries [59]. However, while small numbers do not permit meaningful conclusions to be drawn, our results suggest that more complex systems may perform better than Wigglesworth in at least some developing country settings.

The study teams rated the classifications similarly easy to use despite the differences shown in ratings for retention of information. This may be due to the experience of members of the classification teams that therefore quickly became familiar with the approaches used for the different systems and, if so, this finding may not be replicable in other situations. One might expect that the increased complexity of systems may result in a diminution of user-friendliness. However, we found that the two classifications having the highest number of categories performed best. CODAC may have performed well in terms of ease of use despite far greater numbers of categories due, in part, to the electronic format of this system. CODAC has a user-friendly interface which utilises expandable categories reducing the exposure of the user to the complexity of the system. However, this electronic format may not be suitable in all settings.

Consistency in the application of any classification system is essential. We found inter-rater agreement to be largely suboptimal. Agreement for the major classification categories showed Aberdeen and Wigglesworth perform poorly. ReCoDe was shown to have fair agreement and CODAC and PSANZ-PDC showed good agreement. Tulip performed best with a kappa of 0.74. However, the level of agreement shown is likely to reflect unacceptably low levels of agreement within subcategories even for the best performing system. In assessing agreement, the classifications were applied by individual members of the study team who were not authors of these systems. Publicly available written instructions were provided to the teams, but no other training was provided. The teams agreed that the majority of classification systems failed to provide sufficient instructions on use. While Tulip appeared more difficult to use (according to the *Ease *score), having been used, it performed better in enabling different observers to come to the same conclusions more often than other systems. This suggests that we should examine how Tulip, through its greater focus on pathophysiology, may have important strengths. Agreement may have been higher for all systems if we had used a multidisciplinary panel approach [66]. Classification of stillbirths is often undertaken by individuals without specific training and consequently the finding of suboptimal agreement may reflect reality and thus raises concern about the value of comparisons across and within different settings. The reported agreement for perinatal classifications systems varies. Keeling et al in 1989 [43] reported an 85% agreement with the earlier version of Wigglesworth. Others have reported kappas for major categories ranging from excellent to good: 0.85–0.90 for PSANZ-PDC [11]; 0.81 for Tulip [19]; 0.7 for a classification system by de Galan-Roosen [21,54]; 0.58 for the Fetal and Neonatal Factors (a system based on experience with the Wigglesworth classification); and 0.55 for the Aberdeen system [66]. Many evaluations of classification systems have been undertaken by the original authors or stakeholders themselves. Thus, the potential for systematic bias influencing the conclusions of such studies can not be excluded. While three investigators were closely involved in the development of two of the systems tested (CODAC and PSANZ-PDC) and bias cannot be excluded, steps were taken to minimize this risk and exploration through subgroup analyses, examining scoring for information retention and ease of use, did not reveal any major concerns.

All included systems incorporated some form a hierarchical approach with three systems (Wigglesworth, Aberdeen and ReCoDe) using a more strict approach than the others. One of the perceived benefits of using a hierarchical approach is increased consistency. While small numbers did not permit investigation of this feature, this possible benefit was not apparent in our study. Another benefit of a hierarchical system may be increased user friendliness however this was also not borne out in our study. The danger of strictly hierarchical systems is possible underestimation of the importance of factors further down the list of categories and therefore a loss of focus for research and prevention strategies. If in fact a hierarchical approach confers no benefit in terms of consistency or user friendliness and carries a risk of misleading information on the relative importance of certain factors one could argue that this approach is unwarranted. Further research is required to examine the performance of this approach. While systems which identify all relevant factors about the death are valuable to inform future research and prevention, those which confuse risk factors and associated conditions (e.g. post-maturity, smoking, uncomplicated maternal hypertension, obesity, fetal growth restriction) and/or mechanisms (e.g. "placental insufficiency") with causes of death defeat the main purpose of classifying to identify the underlying cause of death and to allow for future research towards prevention. While we did not specifically evaluate the systems in terms of the interchange between causes and associated conditions, the teams noted that this was an issue for a number of systems particularly for those in which a hierarchical approach is strictly applied. Another important virtue of a classification system is the ability to change over time as disease processes are recognised and better understood. Placental pathology remains an area where much work needs to be done to document the correlation of histological changes on placental function and therefore, ideally, classification systems should allow for expansion in this area.

While the strengths of this study include the large numbers of stillbirths included and the applicability to a wide range of settings, an important limitation was the use of non-validated instruments for assessing the outcome measures of information retention and ease of use. However, while a loss of robustness and diminished validity of our findings as a result of these measurements must be acknowledged, as the instruments were unambiguous in their intent and easy to apply we feel reasonably confident that, for the purposes of our study, they provided useful measures. The subgroup analysis according to stillbirth characteristics conditions [such as stillbirths with fetal growth restriction and placental conditions] was undertaken as these appear to be important clinical groups. However, we recognise that some classification systems are better designed than others to note such conditions leading to an inherent bias in the results.

We did not include the ICD system [10] in our evaluation as it is considered a listing of conditions rather than a classification system per se. The ICD is the international standard diagnostic classification for epidemiological analyses utilising routinely collected data from death certificates and hospitals medical records. There is general consensus amongst those undertaking classification of stillbirth that ICD does not meet their needs. This is largely due to the cumbersome nature of the system as a result of the large number of categories which are not relevant to stillbirth. However, we believe that a link to the ICD system and classifications systems used as a part of clinical audit is crucial for better implementation and further development of definitions of categories. Underpinning the value of any classification system for stillbirth is the collection of adequate and consistent information about these deaths. The current lack of such information is a major barrier to addressing the problem of stillbirths globally. Development and implementation of an internationally accepted minimum dataset and investigation protocol for stillbirths would greatly enhance the value of classifying stillbirths. While data from developing countries in this study were limited, the approach used by the CODAC classification appears promising as an international solution. As reported elsewhere in the BMC [67] the investigator team with additional international collaborators have continued further enhancement of the CODAC system and plan to use this system within their own settings over a period of time prior to re-evaluation of its performance across these diverse settings.

## Conclusion

The basic requirements of stillbirth classification are to retain important information towards understanding the causes of stillbirth, to be easy to apply and have high inter-observer agreement. In this study of six contemporary systems, the Extended Wigglesworth and Amended Aberdeen were clearly shown to perform suboptimally and therefore cannot be recommended for classification of stillbirths. CODAC consistently performed best in terms of retaining important information and ease of use followed by PSANZ-PDC and ReCoDe. Tulip demonstrated the best agreement. All three systems resulted in a low proportion of unexplained stillbirths. Therefore, the virtues of these classifications should be considered in the development of an international classification system. However, further evaluation of the performance of systems in developing country settings is required. Future development of an international solution for classification of stillbirths should strive for alignment in categories and definitions with the ICD system and ensure relevance to developing country settings. Further research is required to better define the placental causes of stillbirth and to identify clinical manifestations of these causes. Measures to ensure consistency in the classification of stillbirths is crucial to undertaking meaningful comparisons across time and geographic locations.

## Competing interests

Three authors were involved in the development of two of the included classification systems: Vicki Flenady in the PSANZ-PDC and CODAC and Frederik Frøen and Halit Pinar in CODAC.

## Authors' contributions

VF developed the study protocol, oversaw the conduct of the study and wrote the manuscript in consultation with FF. All other investigators formed the classification teams and undertook review of stillbirth cases and data collection, interpreted results and edited drafts of the manuscript apart from KG who undertook the data analysis and assisted in interpretation of the results and editing the manuscript. All authors read and approved the final manuscript.

## Pre-publication history

The pre-publication history for this paper can be accessed here:



## Supplementary Material

Additional file 1 **Characteristics of included classification systems**. This file contains a summary of the main characteristics of each of the classification systems included in the study. Click here for file

Additional file 2 **Classification worksheets**. This file contains the classifications categories for each of the classification systems included in the study. Click here for file

Additional file 3 **Unexplained stillbirth by classification and team**. This file shows the frequency distribution for unexplained stillbirths for each classification system across the study teams. Click here for file
