# Supplementary material for: An evaluation of classification systems for stillbirth
Source: BMC Pregnancy Childbirth. 2009 Jun 19;9:24. doi: 10.1186/1471-2393-9-24 (PMC2706223; doi:10.1186/1471-2393-9-24)
Supplement: Additional file 1 — Characteristics of included classification systems. This file contains a summary of the main characteristics of each of the classification systems included in the study. [file 1471-2393-9-24-S1.doc]

Characteristics of included classification systems

| **Classification** | **Country** | **Purpose** | **Intended population** | **Categories**  **Major: Subcategories** | **Hierarchical** | **Factors included** |
| --- | --- | --- | --- | --- | --- | --- |
| Amended Aberdeen (1969) | United Kingdom | To identify the factor that probably initiated the train of events leading to the death for the purpose of prevention | Stillbirth and neonatal death | 8: 22 | Yes | Maternal, fetal |
| Extended Wigglesworth (1986) | United Kingdom | To identify the cause of perinatal death to improve understanding for the purposes of prevention. | Stillbirth and neonatal death | 9:3 | Yes | Maternal, fetal |
| PSANZ-PDC  (Perinatal Society of Australia and New Zealand Perinatal Death Classification) (2004) | Australia and New Zealand | To identify the factor that initiated the sequence of events leading to the death – for the purposes of prevention | Stillbirth and neonatal death | 11:22:53 | Yes, however only strictly applied to the *Congenital Abnormality* category. | Maternal, fetal, limited placental pathology |
| ReCoDe: Relevant conditions and causes of death (2005) | United Kingdom | To identify the relevant condition at the time of death. “What went wrong, ….not necessarily why”. For teaching, counselling, public health policy | Stillbirth and neonatal death | 9:37:2 | Yes | Maternal, fetal, some placental pathology |
| Tulip (2006) | Netherlands | To identify the underlying cause and mechanism of death for the purpose of counselling and prevention | Stillbirth and neonatal death and post neonatal death occurring in the hospital of birth | 6:30:12 | Yes, as a guide only- not intended to be strictly applied. | Maternal, fetal, placental pathology |
| CODAC: Cause of death and associated conditions (2006) | Norway | To enable categorisation of important information to explain stillbirth for the purposes of counselling and prevention through audit, epidemiology and research. | Stillbirth and neonatal death | 10:94:577 | No, apart from terminations of pregnancy. | Maternal, fetal, placental pathology |
