# Supplementary material for: An evaluation of classification systems for stillbirth
Source: BMC Pregnancy Childbirth. 2009 Jun 19;9:24. doi: 10.1186/1471-2393-9-24 (PMC2706223; doi:10.1186/1471-2393-9-24)
Supplement: Additional file 2 — Classification worksheets. This file contains the classifications categories for each of the classification systems included in the study. [file 1471-2393-9-24-S2.doc]

Aberdeen (Amended) Classification Worksheet

| Congenital anomaly | 1. Neural tube defects |
| --- | --- |
|  | 2. Other anomalies |
| Isoimmunisation | 3. Due to rhesus(D) antigen) |
|  | 4. Due to other antigens |
| Pre-eclampsia | 5. Without APH |
|  | 6. Complicated by APH |
| Antepartum Haemorrhage (APH) | 7. With placenta praevia |
|  | 8. With placental abruption |
|  | 9. APH of uncertain origin |
| Mechanical | 10. Cord prolapse or compression with |
|  | 11. Other vertex or face presentation |
|  | 12. Breech presentation |
|  | 13. Oblique or compound presentation , uterine rupture etc. |
| Maternal disorder | 14. Maternal hypertension |
|  | 15. Other maternal disease |
|  | 16. Maternal infection |
| Miscellaneous | 17. Neonatal infection |
|  | 18. Other neonatal disease |
|  | 19. Specific fetal conditions |
| Unexplained | 20. Equal or greater than 2.5kg |
|  | 21. Less than 2.5 Kg |
|  | 22. Unclassifiable |

Cole SK, Hey EN, Thomson AM: **Classifying perinatal death: an obstetric approach**. *Br J Obstet Gynaecol* 1986, **93**(12):1204-1212.

Extended Wigglesworth Classification Worksheet

| 1. Congenital defect/malformation (lethal or sever) |  |
| --- | --- |
| 2. Unexplained antepartum fetal death |  |
| 3. Death from intrapartum asphyxia, anoxia |  |
| 4. Immaturity |  |
| 5. Infection |  |
| 6. Other specific causes | 1. fetal conditions; twin-twin transfusion and hydrops fetalis |
|  | 2. neonatal conditions |
|  | 3. paediatric conditions |
| 7. Accident or non-intrapartum trauma |  |
| 8. Sudden infant death, cause unknown |  |
| 9. Unclassifiable |  |

CESDI - Confidential Enquiry into Stillbirths and Deaths in Infancy: **8th Annual Report**. In*.* London: Maternal and Child Health Research Consortium; 2001.

ReCoDe Classification Worksheet

| A. Fetus | 1. Lethal congenital anomaly |
| --- | --- |
|  | 2. Infection |
|  | 2.1 Chronic – e.g. TORCH |
|  | 2.2 Acute |
|  | 3. Non-immune hydrops |
|  | 4. Iso-immunization |
|  | 5. Fetomaternal hemorrhage |
|  | 6. Twin-twin transfusion |
|  | 7. Intrapartum asphyxia |
|  | 8. Fetal growth restriction^1^ |
|  | 9. Other |
| B. Umbilical Cord | 1. Prolapse |
|  | 2. Constricting loop or knot^2^ |
|  | 3. Velamentous insertion |
|  | 4. Other |
| C. Placenta | 1. Abruptio |
|  | 2. Previa |
|  | 3. Vasa previa |
|  | 4. Placental infarction |
|  | 5. Other placental insufficiency^3^ |
|  | 6. Other |
| D. Amniotic fluid | 1. Chorioamnionitis |
|  | 2. Oligohydramnios^2^ |
|  | 3. Polyhydramnios^2^ |
|  | 4. Other |
| E. Uterus | 1. Rupture |
|  | 2. Uterine anomalies |
|  | 3. Other |
| F. Mother | 1. Diabetes |
|  | 2. Thyroid diseases |
|  | 3. Essential hypertension |
|  | 4. Hypertensive diseases in pregnancy |
|  | 5. Lupus/antiphospholipid syndrome |
|  | 6. Cholestasis |
|  | 7. Drug abuse |
|  | 8. Other |
| G. Trauma | 1. External |
|  | 2. Iatrogenic |
| H. Unclassified | 1. No relevant condition identified |
|  | 2. No information available |

^1^Defined as <10th customized weight-for-gestation percentile

^2^If severe enough to be considered relevant

^3^Histological diagnosis

Gardosi J, Kady SM, McGeown P, Francis A, Tonks A: Classification of stillbirth by relevant condition at death (ReCoDe): population based cohort study. *Bmj* 2005, 331(7525):1113-1117.

PSANZ-PDC Classification Worksheet

| 1. Congenital abnormality (including terminations for congenital abnormalities) | 1.1 Central nervous system |  |
| --- | --- | --- |
|  | 1.2 Cardiovascular system |  |
|  | 1.3 Urinary system |  |
|  | 1.4 Gastrointestinal system |  |
|  | 1.5 Chromosomal |  |
|  | 1.6 Metabolic |  |
|  | 1.7 Multiple/non chromosomal syndromes |  |
|  | 1.8 Other congenital abnormality | 1.81 Musculoskeletal |
|  |  | 1.82 Respiratory |
|  |  | 1.83 Diaphragmatic hernia |
|  |  | 1.84 Hematological |
|  |  | 1.85 Tumors |
|  |  | 1.88 Other specified congenital abnormality |
|  | 1.9 Unspecified congenital abnormality |  |
| 2. Perinatal infection | 2.1 Bacterial | 2.11 Group B Streptococcus |
|  |  | 2.12 E coli |
|  |  | 2.13 Listeria monocytogenes |
|  |  | 2.14 Spirochaetal e.g. Syphilis |
|  |  | 2.18 Other bacterial |
|  |  | 2.19 Unspecified bacterial |
|  | 2.2 Viral | 2.21 Cytomegalovirus |
|  |  | 2.22 Parvovirus |
|  |  | 2.23 Herpes simplex virus |
|  |  | 2.24 Rubella virus |
|  |  | 2.28 Other viral |
|  |  | 2.29 Unspecified viral |
|  | 2.3 Protozoal e.g. Toxoplasma |  |
|  | 2.5 Fungal |  |
|  | 2.8 Other specified organism |  |
|  | 2.9 Other unspecified organism |  |
| 3. Hypertension | 3.1 Chronic hypertension: essential |  |
|  | 3.2 Chronic hypertension: secondary, e.g. renal disease |  |
|  | 3.3 Chronic hypertension: unspecified |  |
|  | 3.4 Gestational hypertension |  |
|  | 3.5 Pre-eclampsia | 3.51 With laboratory evidence of thrombophilia |
|  | 3.6 Pre-eclampsia superimposed on chronic hypertension | 3.61 With laboratory evidence of thrombophilia |
|  | 3.9 Unspecified hypertension |  |
| 4. Antepartum hemorrhage (APH) | 4.1 Placental abruption | 4.11 With laboratory evidence of thrombophilia |
|  | 4.2 Placenta praevia |  |
|  | 4.3 Vasa praevia |  |
|  | 4.8 Other APH |  |
|  | 4.9 APH of undetermined origin |  |
| 5. Maternal conditions | 5.1 Termination of pregnancy for maternal psychosocial indications |  |
|  | 5.2 Diabetes / Gestational diabetes |  |
|  | 5.3 Maternal injury | 5.31 Accidental |
|  |  | 5.32 Non-accidental |
|  | 5.4 Maternal sepsis |  |
|  | 5.5 Lupus obstetric syndrome |  |
|  | 5.6 Obstetric cholestasis |  |
|  | 5.8 Other specified maternal conditions |  |
| 6. Specific perinatal conditions | 6.1 Twin-twin transfusion |  |
|  | 6.2 Fetomaternal hemorrhage |  |
|  | 6.3 Antepartum cord complications (e.g. cord hemorrhage; true knot with evidence of occlusion) |  |
|  | 6.4 Uterine abnormalities, e.g. bicornuate uterus, cervical incompetence |  |
|  | 6.5 Birth trauma (typically infants of >24 weeks gestation or >600g birthweight) |  |
|  | 6.6 Alloimmune disease | 6.61 Rhesus |
|  |  | 6.62 ABO |
|  |  | 6.63 Kell |
|  |  | 6.64 Alloimmune thrombocytopenia |
|  |  | 6.68 Other |
|  |  | 6.69 Unspecified |
|  | 6.7 Idiopathic hydrops |  |
|  | 6.8 Other specific perinatal conditions (includes iatrogenic conditions such as rupture of membranes after amniocentesis, termination of pregnancy for suspected but unconfirmed congenital abnormality). |  |
| 7. Hypoxic peripartum death (typically infants of >24 weeks gestation or >600g birthweight) | 7.1 With intrapartum complications | 7.11 Uterine rupture |
|  |  | 7.12 Cord prolapse |
|  |  | 7.13 Shoulder dystocia |
|  |  | 7.18 Other |
|  | 7.2 Evidence of non-reassuring fetal status in a normally grown infant (e.g. abnormal fetal heart rate, fetal scalp pH/lactate, fetal pulse oximetry without intrapartum complications) |  |
|  | 7.3 No intrapartum complications and no evidence of non-reassuring fetal status. |  |
|  | 7.9 Unspecified hypoxic peripartum death |  |
| 8. Fetal Growth Restriction (FGR) | 8.1 With evidence of reduced vascular perfusion on Doppler studies and /or placental histopathology (e.g. significant infarction, acute atherosis, maternal and/or fetal vascular thrombosis or maternal floor infarction) |  |
|  | 8.2 With chronic villitis |  |
|  | 8.3 No placental pathology |  |
|  | 8.4 No examination of placenta |  |
|  | 8.8 Other specified placental pathology |  |
|  | 8.9 Unspecified or not known whether placenta examined |  |
| 9. Spontaneous preterm (<37 weeks gestation) | 9.1 Spontaneous preterm with intact membranes, or membrane rupture <24 hours before delivery | 9.11 With chorioamnionitis on placental histopathology |
|  |  | 9.12 Without chorioamnionitis on placental histopathology |
|  |  | 9.13 With clinical evidence of chorioamnionitis, no examination of placenta |
|  |  | 9.17 No clinical signs of chorioamnionitis, no examination of placenta |
|  |  | 9.19 Unspecified or not known whether placenta examined |
|  | 9.2 Spontaneous preterm with membrane rupture ³24 hours before delivery | 9.21 With chorioamnionitis on placental histopathology |
|  |  | 9.22 Without chorioamnionitis on placental histopathology |
|  |  | 9.23 With clinical evidence of chorioamnionitis, no examination of placenta |
|  |  | 9.27 No clinical signs of chorioamnionitis, no examination of placenta |
|  |  | 9.29 Unspecified or not known whether placenta examined |
|  | 9.3 Spontaneous preterm with membrane rupture of unknown duration before delivery | 9.31 With chorioamnionitis on placental histopathology |
|  |  | 9.32 Without chorioamnionitis on placental histopathology |
|  |  | 9.33 With clinical evidence of chorioamnionitis, no examination of placenta |
|  |  | 9.37 No clinical signs of chorioamnionitis, no examination of placenta |
|  |  | 9.39 Unspecified or not known whether placenta examined |
| 10. Unexplained antepartum death | 10.1 With evidence of reduced vascular perfusion on Doppler studies and /or placental histopathology (e.g. significant infarction, acute atherosis, maternal and/or fetal vascular thrombosis or maternal floor infarction) |  |
|  | 10.2 With chronic villitis |  |
|  | 10.3 No placental pathology |  |
|  | 10.4 No examination of placenta |  |
|  | 10.8 Other specified placental pathology |  |
|  | 10.9 Unspecified or not known whether placenta examined |  |
| 11. No obstetric antecedent | 11.1 Sudden Infant Death Syndrome (SIDS) | 11.11 SIDS Category IA: Classic features of SIDS present and completely documented |
|  |  | 11.12 SIDS Category IB: Classic features of SIDS present but incompletely documented |
|  |  | 11.13 SIDS Category II : Infant deaths that meet Category I except for one or more features |
|  | 11.2 Postnatally acquired infection |  |
|  | 11.3 Accidental asphyxiation |  |
|  | 11.4 Other accident, poisoning or violence (postnatal) |  |
|  | 11.8 Other specified |  |
|  | 11.9 Unknown/Undetermined | 11.91 Unclassified Sudden Infant Death |
|  |  | 11.92 Other Unknown/Undetermined |

Chan A, King JF, Flenady V, Haslam RH, Tudehope DI: Classification of perinatal deaths: development of the Australian and New Zealand classifications. *J Paediatr Child Health* 2004, 40(7):340-347.

Perinatal Mortality Special Interest Group of the Perinatal Society of Australia and New Zealand: **Clinical Practice Guideline for perinatal mortality audit**. In*.* Brisbane, Australia: Perinatal Society of Australia and New Zealand; 2004. http://www.psanzpnmsig.org/
