# Supplementary material for: An evaluation of classification systems for stillbirth
Source: BMC Pregnancy Childbirth. 2009 Jun 19;9:24. doi: 10.1186/1471-2393-9-24 (PMC2706223; doi:10.1186/1471-2393-9-24)
Supplement: Additional file 3 — Unexplained stillbirth by classification and team. This file shows the frequency distribution for unexplained stillbirths for each classification system across the study teams. [file 1471-2393-9-24-S3.doc]

**Unexplained stillbirth by classification and team**

|  |  | **Classification system** | | | | | | | | | | | | | |
| --- | --- | --- | --- | --- | --- | --- | --- | --- | --- | --- | --- | --- | --- | --- | --- |
| **Team** | **Total Cases**  **n** | **Wigglesworth** | | **Aberdeen** | | **PSANZ-PDC** | | **PSANZ-PDC*** | | **ReCoDe** | | **Tulip** | | **CODAC** | |
|  |  | n | % | n | % | n | % | n | % | n | % | n | % | n | % |
| 1 | 100 | 53 | 53.0 | 29 | 29.0 | 38 | 38.0 | 6 | 6.0 | 11 | 11.0 | 10 | 10.0 | 5 | 5.0 |
| 2 | 102 | 60 | 58.8 | 41 | 40.2 | 27 | 26.5 | 10 | 9.8 | 2 | 2.0 | 6 | 5.9 | 3 | 2.9 |
| 3 | 106 | 22 | 20.8 | 6 | 5.7 | 2 | 1.9 | 2 | 1.9 | 7 | 6.6 | 3 | 2.8 | 16 | 15.1 |
| 4 | 101 | 51 | 50.5 | 37 | 36.6 | 33 | 32.7 | 19 | 18.8 | 23 | 22.8 | 14 | 13.9 | 10 | 9.9 |
| 5 | 100 | 57 | 57.0 | 56 | 56.0 | 35 | 35.0 | 26 | 26.0 | 30 | 30.0 | 27 | 27.0 | 29 | 29.0 |
| 6 | 100 | 85 | 85.0 | 66 | 66.0 | 31 | 31.0 | 11 | 11.0 | 17 | 17.0 | 15 | 15.0 | 16 | 16.0 |
| 7 | 67 | 24 | 35.8 | 25 | 37.3 | 6 | 9.0 | 42 | 62.7 | 3 | 4.5 | 1 | 1.5 | 1 | 1.5 |
| 8 | 95 | 35 | 36.8 | 46 | 48.4 | 34 | 35.8 | 15 | 15.8 | 19 | 20.0 | 11 | 11.6 | 1 | 1.1 |
| 9 | 86 | 43 | 50.0 | 74 | 86.0 | 42 | 48.8 | 1 | 1.2 | 6 | 7.0 | 0 | 0.0 | 0 | 0.0 |
|  | **857** | 430 | **50.2** | 380 | **44.3** | 248 | **28.9** | 132 | **15.4** | 118 | **13.8** | 87 | **10.2** | 81 | **9.5** |

Classification categories included: Wigglesworth 2; Aberdeen 20, 21; PSANZ-PDC 10 (including all subcategories), and *****10.3 (i.e. excluding placental pathology subcategories); ReCode H1; Tulip 6.1; CODAC 811.
